# Training in the use of the water jet and cold atmospheric plasma jet for the decontamination of dental implants

**DOI:** 10.1007/s00784-024-05749-5

**Published:** 2024-06-04

**Authors:** Rutger Matthes, Lukasz Jablonowski, Vinay Pitchika, Birte Holtfreter, Christian Eberhard, Torsten Gerling, Juliane Wagner, Christian Flörke, Anne-Katrin Eisenbeiß, Raluca Cosgarea, Karin Jepsen, Jennifer Bunke, Ausra Ramanauskaite, Amira Begić, Karina Obreja, Maria Mksoud, Thomas Kocher

**Affiliations:** 1 Department of Restorative Dentistry, Periodontology, Endodontology, Preventive Dentistry and Pedodontics, Dental School, University Medicine Greifswald, Fleischmannstr. 42, 17475 Greifswald, Germany; 2 Department of Oral Diagnostics, Digital Health and Health Services Research, Charité—Universitätsmedizin Berlin, Aßmannshauser Str. 4-6, 14197 Berlin, Germany; 3 Sirona Dental Systems GmbH, Bensheim, Germany; 4 Leibniz-Institute for Plasma Science and Technology e.V. (INP), ZIK plasmatis, Greifswald, Germany; 5 Department of Oral and Maxillofacial Surgery, Christian-Albrechts University Kiel, Kiel, Germany; 6 Department of Periodontology, Operative and Preventive Dentistry, University of Bonn, Bonn, Germany; 7 Clinic for Periodontology and Peri-implant Diseases, University of Marburg, Marburg, Germany; 8 Iuliu-Hatieganu University of Medicine and Pharmacy, Cluj-Napoca, Romania; 9 Department of Oral Surgery and Implantology, Johann Wolfgang Goethe-University, Carolinum, Frankfurt, Germany; 10 Department of Oral and Maxillofacial Surgery/Plastic Surgery, University Medicine Greifswald, Greifswald, Germany

**Keywords:** Artificial plaque, User training, Water jet, Dental implants, peri-implantitis, Cold plasma

## Abstract

**Objectives:**

Clinical trials testing new devices require prior training on dummies to minimize the "learning curve" for patients. Dentists were trained using a novel water jet device for mechanical cleaning of dental implants and with a novel cold plasma device for surface functionalisation during a simulated open flap peri-implantitis therapy. The hypothesis was that there would be a learning curve for both devices.

**Materials and methods:**

11 dentists instrumented 44 implants in a dummy-fixed jaw model. The effect of the water jet treatment was assessed as stain removal and the effect of cold plasma treatment as surface wettability. Both results were analysed using photographs. To improve treatment skills, each dentist treated four implants and checked the results immediately after the treatment as feedback.

**Results:**

Water jet treatment significantly improved from the first to the second implant from 62.7% to 75.3% stain removal, with no further improvement up to the fourth implant. The wettability with cold plasma application reached immediately a high level at the first implant and was unchanged to the 4th implant (mean scores 2.7 out of 3).

**Conclusion:**

A moderate learning curve was found for handling of the water jet but none for handling of the cold plasma**.**

**Clinical relevance:**

*Scientific rational for study:* Two new devices were developed for peri-implantitis treatment (Dental water jet, cold plasma). Dentists were trained in the use of these devices prior to the trial to minimize learning effects.

*Principal findings:* Experienced dentists learn the handling of the water jet very rapidly and for cold plasma they do not need much training.

*Practical implications:* A clinical study is in process. When the planned clinical study will be finished, we will find out, if this dummy head exercise really minimised the learning curve for these devices.

## Introduction

Training in the handling and use of medical devices is necessary for undergraduates or residents to acquire clinical skills [1, 2], but experts should also practise with new medical devices before using them on patients. When multicentre clinical trials with new devices are planned, in addition to standardised outcome assessment by blinded examiners [3], it is essential that the operators involved should use the devices ideally in standardised high quality level. Surgical procedure or skill training using dummies in a simulated environment that is close to real clinical situation is fundamental to achieve optimal results among examiners [4].

In many surgical specialities, hands-on training on dummies, cadaver or in virtual reality is a part of the student, residency or continuing education programs to learn specific aspects of surgical procedures or to acquire technical skills to handle a specific device [3, 5]. If the training provides an objective outcome, continuous feedback loops of the achieved outcomes give the trainee the opportunity to reflect on his performance and to improve his skills and the trainer could easily communicate improvement to the trainee. Repeated training could lead to an increased performance because the trainee better understands the restriction and inherent difficulties of the training tasks [1, 6, 7].

Current surgical therapy of peri-implantitis has uncertain success in arresting or reversing the progression peri-implantitis [8], because no mechanical decontamination method can yet guarantee a sufficient biofilm removal [9]. The dental community needs new decontamination methods for the treatment of peri-implantitis. In an interdisciplinary process, our laboratory has been working on cold atmospheric pressure plasma devices (CAP) for peri-implantitis therapy for more than 10 years [10, 11]. Finally a combined treatment method was developed based on a Dental water jet and a CAP device, which have been successfully tested in vitro and described in detail elsewhere [10]. The Dental water jet removes plaque and the CAP device kills residual bacteria, removes bacterial by-products and hydrophilizes the surface. In order to conduct a multicentre clinical trial (DRKS-ID: DRKS00026673)[12], the operating dentists were trained in the use of the Dental water jet and the CAP device prior to the begin of the trial to avoid or at least minimise learning effects during the clinical RCT that would reduce the quality of the results. The hypothesis was that there would be a learning curve for treatment with Dental water jet and CAP. Furthermore, depending on the results, the trainee would have to be retrained if an a priori determined performance threshold for each device was not exceeded.

## Material and methods

### Treatment simulation and test conditions

**Fig. 1:**
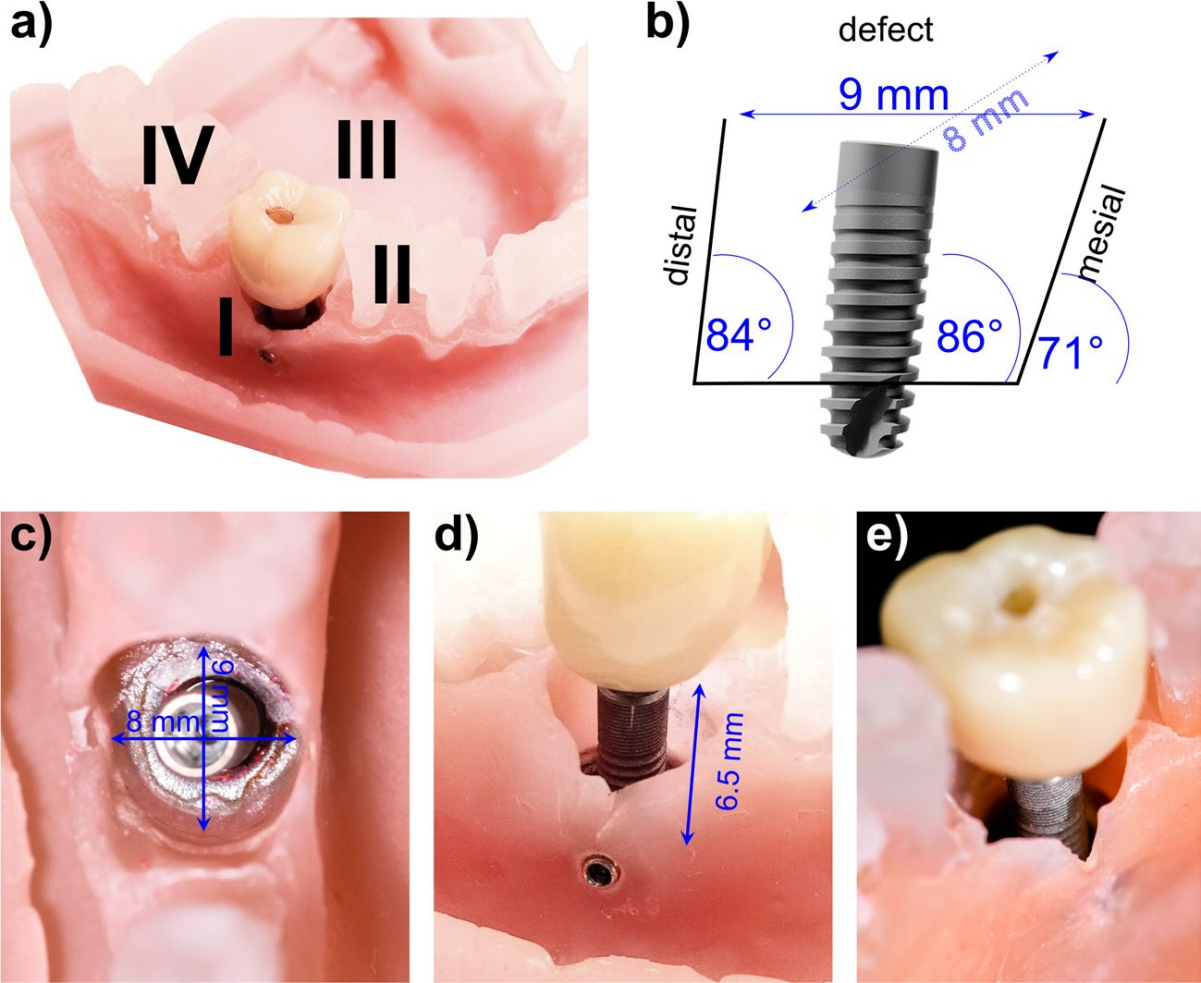
The jaw model and the dimension of the peri-implant defect for training. Image **a**) shows the position of the defect with implant and crown with the four implant sites to be analysed (I) buccal, II) mesial, III) lingual, IV) distal; **b**) shows the dimensions of the defect in mesio-distal direction; **c**) shows the defect from an occlusal aspect; **d**) shows the defect with implant and crown from buccal aspect; **e**) shows the defect with implant and crown form the lingual aspect

A dummy head for student training was attached to the dental chair and a mandible was fabricated from a cast of a real patient. A crater-shaped circumferential peri-implantitis defect was created at position 46 (FDI World Dental Federation (ISO) notation). Bone-level implants (length of 11 mm, diameter of 3.6 mm, OsseoSpeed™ EV 3.6S-11mm, Dentsply Sirona, Hanau, Germany) were used, fixed with a transversal screw in the dummy mandible (Fig. 1a). After each treatment, the implants were unscrewed and replaced with new implants. The coronal width of the defect varied between 8 mm (buccal-lingual) and 9 mm (distal-mesial) and the vertical height of the defect was approximately 6 mm (Fig. 1b-d). The implant with the crown was fixed at an angle of 86.2°, exposing 6.5 mm of implant surface (corresponding to 4 threads, called treatable implant area), and 4.5 mm of the implant was embedded in the dummy mandible (Fig. 1b,d). The whole circumferential area was considered as treatable, but with varying degrees of complexity. Since no prosthetic restorations are to be removed in the planned RCT, the screw-retained crown on the implant was not removed to hamper access to “exposed” implant surfaces (Fig. 1a,e).

Eleven experienced dentists (5 oral surgeons, 4 periodontists, 2 maxillofacial surgeons, professional experience between 28 and 7 years (mean 11.6 years), one male, 10 females, from the dental schools of Greifswald, Kiel, Bonn and Frankfurt, Germany) participated in the training sessions with an assistant, who aspirated the water spray of the Dental water jet or the ozone exhaust of CAP from the manikin`s oral cavity. First the Dental water jet treatment was performed on four implants, then followed by the CAP treatment with further four implants (11 trainees × 4 implants × 2 devices = 88 implants). As in the planned RCT, the treatment time was limited to a total of 120 s for both Dental water jet and CAP, corresponding to 60 s per buccal or lingual access. The treatment time is based on former in-vitro studies with these devices [13] and on the experience with this jaw model. After each treatment, the implant was removed from the jaw model and the trainee was able to view where the stain was not removed after the waterjet treatment or where the plasma plume did not hydrophilize the surface after CAP treatment. In this way the trainee gained a deeper understanding of how to use the CAP and how to angle the nozzles of the water jet to improve his performance. A priori, based on decontamination results during pre-tests on discs with artificial plaque and *in-vitro* multispecies-biofilm, a threshold was set ≥ 70% for stain removal and ≥ 75% for wettability as means of the four runs. Wettability ≥ 75% corresponds to 9 out of 12 drops spreading on one implant. If a participating dentist did not exceed these thresholds, the training was repeated at least one month later. Only the results of the first training sessions were evaluated for this publication.

### Dental water jet treatment and analysis

**Fig. 2:**
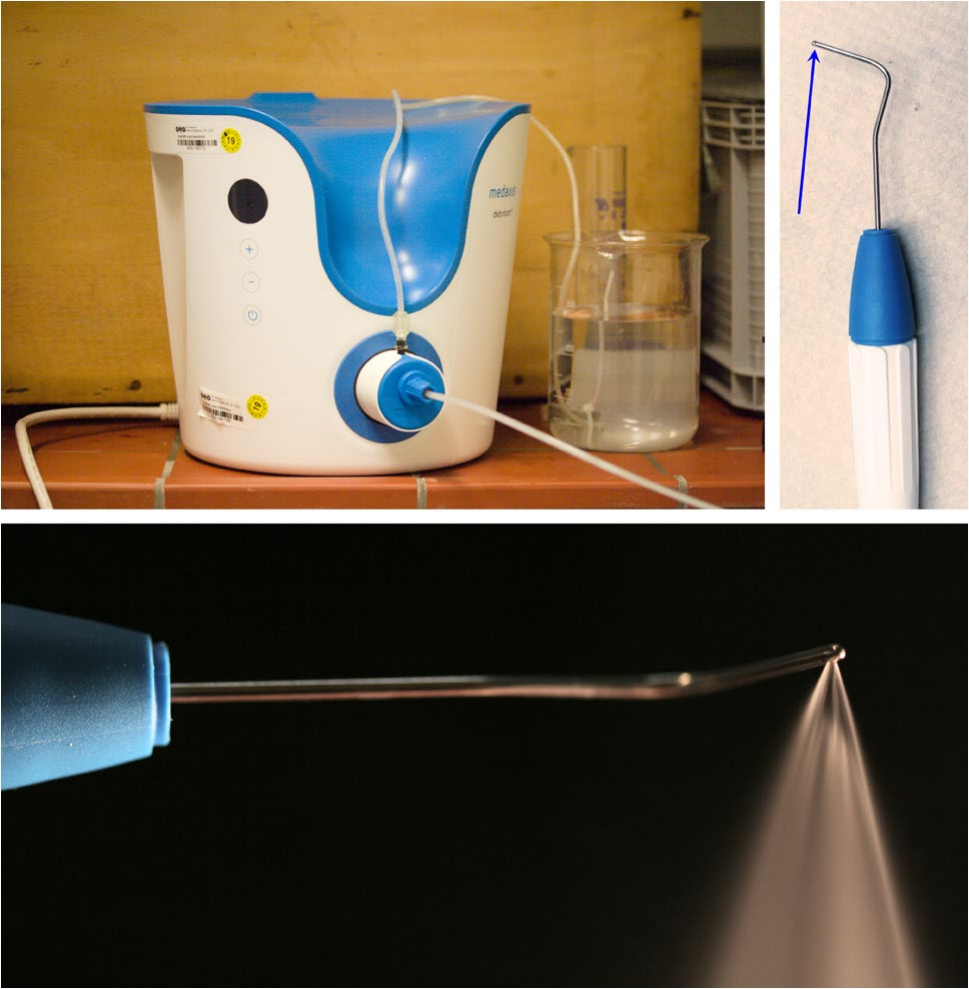
Dental water jet device (top row left) with handpiece (top row right), the arrow shows the nozzle outlet for the right-side application. The lower row shows the right sided water spray

The Dental water jet is based on the debritom + (Swiss company Medaxis AG, Baar, Switzerland), which is CE-certified for cleaning, irrigating and debriding wounds and other skin diseases. The newly developed water jet handpiece has angled application tips with a right and a left side water outlet (Sirona Dental Systems (Dentsply Sirona, Bensheim, Germany) with sterile isotonic saline solution as irrigation medium (ISS, 0.9%, Fresenius Kabi, Bad Homburg, Germany) (Fig. 2). The treatment was performed at intensity level 3 of debritom + , corresponding to a flow rate of 72 ± 2 ml/min.

**Fig. 3:**
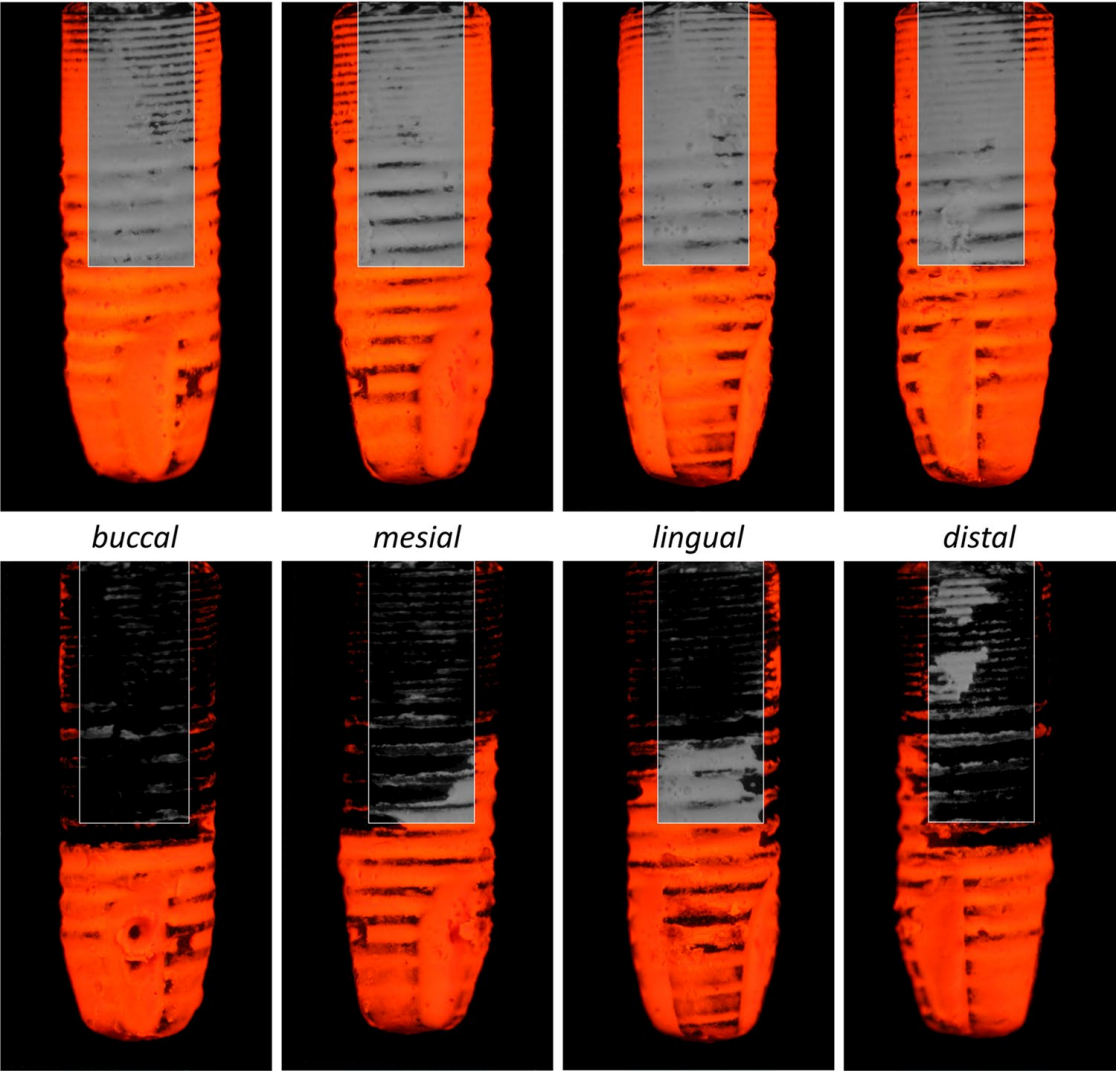
Examples of implants stained with artificial plaque before (top row) and after Dental water jet treatment (bottom row) for the buccal, mesial, lingual, and distal site under UV-A light. The rectangular areas of interest used for image analysis are overlaid. Grey represents untreated area within the area of interest

For the assessment of the water jet treatment, the implants (N = 11 × 4) were stained with an artificial, water insoluble plaque (Nissin Dental Products Inc., Tokyo, Japan). Before and after treatment, photographs of the stained implants against a black background were taken from four sies (buccal, mesial, lingual, and distal) in identical views using a standardised procedure with a digital camera (EOS 450D, Canon Inc., Tokyo, Japan) with a 60-mm lens (Canon Inc., Tokyo, Japan). All photos were taken with the following settings: fixed white balance, ISO 100, f/8 aperture, shutter speed 1/10 s. Two UV-A lamps (LED light, 395 nm) were used as light source at a distance of approximately 4 cm. An orange filter was used to separate the intense orange-red fluorescence of the artificial plaque from other light waves to obtain a very high contrast (Fig. 3).

These coloured images were converted to greyscale images. The grey values were evaluated for the region of interest corresponding to the treatable implant area (small threads and first 4 main threads) (Fig. 3) by using ImageJ (v1.50, US National Institutes of Health, Bethesda, MD, USA).

### Cold plasma treatment and analysis

**Fig. 4:**
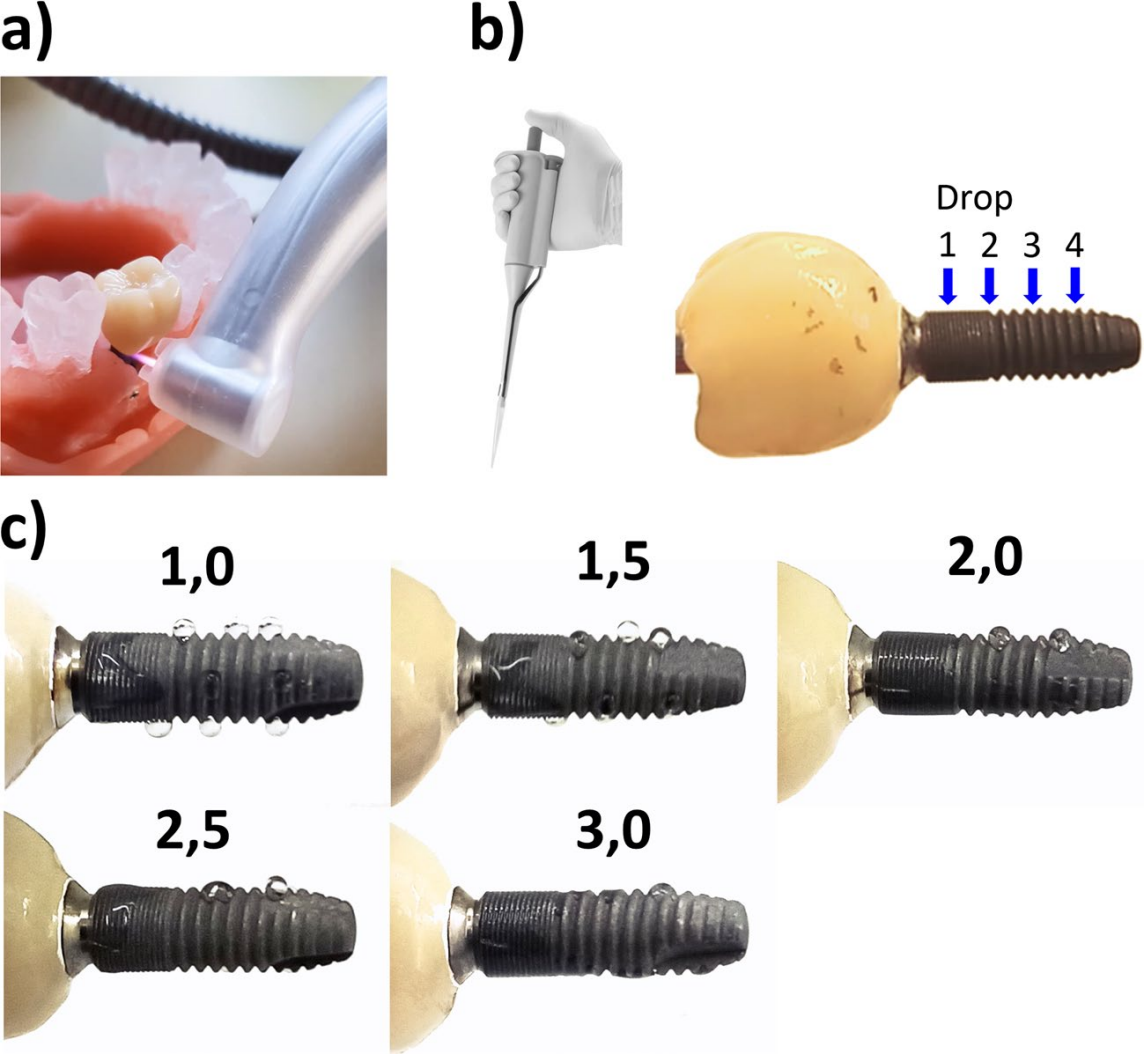
Scoring of CAP treatment; **a**) CAP on the implant in the jaw model; **b**) a pipette was used to drop 5 µl of distilled water on the upper implant side at four positions (depicted by arrows); **c**), sum wettability scores between 1 and 3 was given to evaluate the CAP hydrophilicity effect, drop at position 4 represents untreated control position

A cold atmospheric pressure plasma (CAP) device was used, developed by the Leibniz Institute for Plasma Science and Technology (INP), Greifswald, Germany. It is an argon-based plasma jet working at a frequency of 0.95 MHz at 2–3 kVpp and having maximum DC-power input of 1.6 W. The noble gas argon (99,999%, ALPHAGAZ, Air Liquide, Düsseldorf, Germany) was used as the carrier gas with a flow rate of 2.3 slm (standard litre per minute) set on the CAP device. The plasma technology was incorporated into a commercially available dental contra-angle handpiece (Fig. 4a).

The effect of CAP treatment was verified by its ability to hydrophilize the hydrophobic surface of the rough titanium implant. To assess the hydrophilicity, the implant (N = 11 × 4) was aligned horizontally. Four drops of distilled water (5 µl) were pipetted onto the upper side of the implant in line at a distance of approximately 2 mm (Fig. 4b,c). The spread of the first three water drops was scored from 0 to 3. The wettability score 0: drop remained spherical; score 1: only the first drop spread; score 2: the first two drops spread; score 3: the first three drops were spread; 0.5 steps were given, if the water drop spreading was visible but not completed (Fig. 4c). The fourth drop served as a control for the initial hydrophobicity of the implant, as the apical threads could not be reached by the plasma plume, because they anchored the implant in the jaw model. After evaluating one surface, the implant was rotated by 90° to repeat the evaluation for all four implant surfaces.

### Statistical analyses

Means with standard deviations (SD) and numbers (percentages) were reported for continuous and categorical variables, respectively. Linear models were applied using generalised least squares to estimate effects of implant (1–4), implant surface (mesial, buccal, distal, lingual), treatment centre (Bonn, Frankfurt am Main, Greifswald, Kiel), stain thickness (weak, well), and light exposure (reduced, well) on change in mean grey value of the image, and effects of implant (1–4), implant surface (mesial, buccal, distal, lingual), and treatment centre (Bonn, Frankfurt am Main, Greifswald, Kiel) on change of the wettability score. Models were repeated by surface (mesial, buccal, distal, lingual). Linear regression coefficients (Beta), 95% confidence intervals (CI) and p values were reported.

Significance was set at q < 0.05. All analyses were performed using Stata/SE Version 17.0 [14] and R 4.2.1 [15].

## Results

### Artificial plaque removal by Dental water jet treatment

**Fig. 5:**
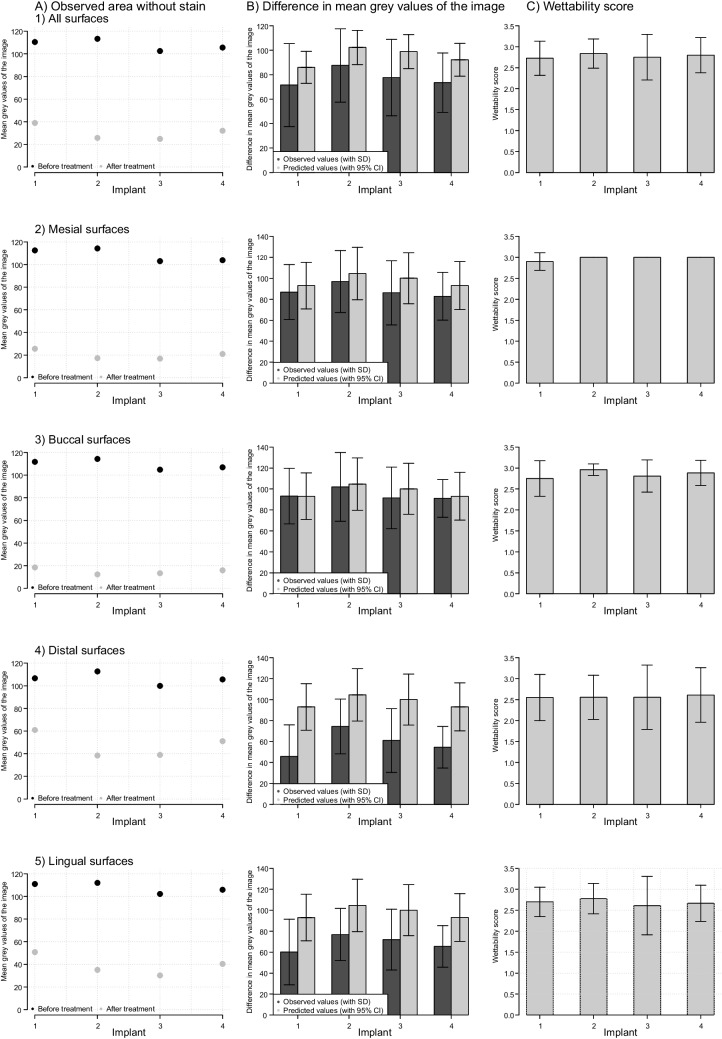
Observed mean grey values (± standard deviation) before and after dental water jet treatment (column **A**) and observed and predicted (by the generalised least square linear regression models from Table 1) changes in mean grey values (column **B**) of the analysed images, as well as wettability scores after cold plasma treatment (column **C**) by implant number (x axis) including all surfaces (row 1) or stratifying by surface (rows 2–5)

The lowest amount of stain was removed from implant 1 by all dentists. The observed values already showed that the treatment of the 2nd implant in particular led to a significant improvement in cleaning, while this decreased again slightly for the 3rd and 4th implants. The optimisation was most pronounced in the cleaning of the distal and lingual implant surfaces (Fig. 5A and B). However, it can also be seen that the observed values taken before treatment fluctuate. In order to take into account the influencing variables on the measurement mentioned in the methodology, a statistical model was adapted accordingly (Appendix Table 3). Thus, implant 1 served as a comparison basis for the statistical model. The following results are derived from this. From the 1st to the 2nd and 3rd implant the area without stain significantly increased by 16.25 and 12.94 grey values on average (p < 0.01), respectively, whereas the treatment of 4th implant was less effective than the two previous treatments (average stain removal was 6.17 grey values) (Table 1) corresponding to 72% of stain removal for implant 4, whereas the dye removal was measured at 74.7% for implant 3, 75.3% for implant 2 and 62.7% for implant 1. This pattern of a sharp increase between the first and second implant, a virtually unchanged performance on the third implant, and a decrease to the values obtained on the first implant was observed for all surfaces (Fig. 5B). Irrespective of the treated implant, the buccal surface was the easiest to clean (6.47 higher mean grey value compared to mesial surfaces). For distal and lingual surfaces, mean grey values were 29.26 and 19.42 lower compared to mesial surfaces; values are indicative of significantly more stain left compared to the mesial surface, adjusted for implant number (Table 1). The pattern of a sharp increase between the first and second implant was most pronounced for the lingual and distal surfaces (mean grey values were 17.57 and 28.96 higher for implant 2 compared to implant 1, respectively) (Table 1).

**Table 1:** Results from generalised least square linear regression models regressing change in mean grey values of the image on implant and surface (upper part) or on implant only for specific surfaces (bottom part). All models additionally included the following covariates: stain thickness, light exposure, and treatment centre

Variable	Category	Beta (95% C.I.)	*P* value
*All surfaces*			
Implant (Ref.: Implant 1)	Implant 2	16.25 (9.17; 23.32)	< 0.01
	Implant 3	12.94 (5.82 20.07)	< 0.01
	Implant 4	6.17 (-0.90; 13.24)	0.09
Surface (Ref.: Mesial)	Buccal	6.47 (-0.47; 13.40)	0.07
	Distal	-29.26 (-36.20; -22.33)	< 0.01
	Lingual	-19.42 (-26.34; -12.51)	< 0.01
*Stratified by surface*			
Buccal surfaces (Implant side I)			
Implant (Ref.: Implant 1)	Implant 2	6.56 (-4.75; 17.88)	0.26
	Implant 3	3.18 (-8.02; 14.38)	0.58
	Implant 4	-0.37 (-11.66; 10.92)	0.95
Mesial surfaces (Implant side II)			
Implant (Ref.: Implant 1)	Implant 2	11.55 (-1.01; 24.11)	0.08
	Implant 3	7.05 (-5.32; 19.42)	0.27
	Implant 4	0.05 (-12.12; 12.21)	0.99
Lingual surfaces (Implant side III)			
Implant (Ref.: Implant 1)	Implant 2	17.57 (1.53; 33.61)	0.04
	Implant 3	20.31 (4.20; 36.42)	0.02
	Implant 4	11.18 (-4.66; 27.02)	0.18
Distal surfaces (Implant side IV)			
Implant (Ref.: Implant 1)	Implant 2	28.96 (13.83; 44.08)	< 0.01
	Implant 3	21.56 (6.02; 37.11)	0.01
	Implant 4	12.82 (-2.57; 28.20)	0.11

Three trainees did not exceed the previously set threshold of 70% and had to repeat their training.

### Hydrophilicity by CAP treatment

Already on the first treated implant, a mean wettability score of 2.7 was achieved. The mean scores did not significantly vary in the following 3 treated implants (Fig. 5C, Table 2). The lingual and the distal surfaces had lower mean wettability scores (2.5–2.8) than the buccal and mesial surfaces (2.7–3.0) (Fig. 5C). In the apical direction on the implant, the scores were lower. The most coronal drop spread in almost all cases (99%) (score > 0.5). In 95% of the cases, the second droplet also spread completely (score > 1.5). In the third, most apical drop, 89% of the implants were still wettable after plasma treatment (score > 2), and complete spreading of all three drops was achieved in 73% of the treated implants (score > 2.5). (Appendix Table 4). The fourth drop (negative control) always remained as a drop on a hydrophobic surface.

**Table 2:** Results from generalised least square linear regression models regressing the wettability score after CAP treatment either on implant and surface (upper part) or on implant only for specific surfaces (bottom part). All models included also treatment centre as a covariate

Variable	Category	Beta (95% CI)	*P* value
*All surfaces*			
Implant (Ref.: Implant 1)	Implant 2	0.102 (-0.062; 0.267)	0.23
	Implant 3	0.015 (-0.150; 0.179)	0.86
	Implant 4	0.065 (-0.100; 0.229)	0.44
Surface (Ref.: Mesial)	Buccal	-0.094 (-0.256; 0.068)	0.26
	Distal	-0.405 (-0.576; -0.235)	< 0.01
	Lingual	-0.284 (-0.455; -0.113)	0.00
*Stratified by surface*			
Buccal surfaces (Implant side I)			
Implant (Ref.: Implant 1)	Implant 2	0.248 (0.005; 0.491)	0.05
	Implant 3	0.094 (-0.149; 0.338)	0.45
	Implant 4	0.171 (-0.072; 0.415)	0.18
Mesial surfaces (Implant side II)			
Implant (Ref.: Implant 1)	Implant 2	0.098 (0.002; 0.194)	0.06
	Implant 3	0.098 (0.002; 0.194)	0.06
	Implant 4	0.098 (0.002; 0.194)	0.06
Lingual surfaces (Implant side III)			
Implant (Ref.: Implant 1)	Implant 2	0.058 (-0.284; 0.401)	0.74
	Implant 3	-0.109 (-0.451; 0.234)	0.54
	Implant 4	-0.053 (-0.395; 0.289)	0.76
Distal surfaces (Implant side IV)			
Implant (Ref.: Implant 1)	Implant 2	0.005 (-0.517; 0.526)	0.99
	Implant 3	0.005 (-0.517; 0.526)	0.99
	Implant 4	0.060 (-0.462; 0.582)	0.82

All trainees reached a mean wettability score between 2.3 (77%) and 3.0 (100%), which was above the previously set threshold of 75%.

## Discussion

For the Dental Water jet the hypothesis was confirmed, that the training led to improved handling, but not for the CAP treatment. Stain removal increased statistically significantly from 62.7% to 75.3% from the 1st to the 2nd implant, whereas 3rd treatment did not improve the performance (stain removal 74.7%). The treatment of 4th implant even led to less favourable results with a difference to the first implant of only 6.17 grey levels (≙ 69.5% stain removal). Only three trainees did not exceed the prior determined threshold of 70%. We assume that the first visual feedback gave the most important impulse to adjust the handling of the Dental water jet. The lack of change from the second to third implant could be due to the fact that the trainee did not learn anymore from his inspection, because the handling of this water jet device is very similar to an air-powder instrument, with which dentists have been familiar for decades. The relapse from the treatment of the third to the fourth implant could be explained by the fact, that the trainee no longer found the task challenging—his attention and motivation waned. He wanted to finish the training sessions and felt confident enough to have cleaned the implant. Motivation has a big impact on performance, which was elegantly demonstrated in a dummy head trial with dental students [16]. One group was praised for their progress and received incentives, if they achieved certain targets, while the other group received normal attention from their teachers. The highly motivated group removed 84.9% of stain whereas the other groups removed on average 71%. Taken together these *in-vitro* results mean, that only one training session is required for experienced trainees with the Dental water jet.

Depending on technical parameters, plasma can have various effects on eukaryotic cells (e.g. growth stimulation, wound healing, tumour necrosis), inactivate prokaryotic cells (mainly through membrane damage) or clean surfaces or generate changes in charge and hydrophilicity on surfaces [17–19]. The generation of hydrophilic surfaces, which is made possible by the source used, was utilised to evaluate the propagation of the plasma on the implant in order to assess the success of the training. For the CAP, the hypothesis, that the training leads to improved handling could not be confirmed as the trainees already achieved very high wettability scores of around 2.7 out of 3 on the first implant, which did not change significantly on the following three treated implants indicating easy and appropriate handling of the plasma jet handpiece. All trainees exceeded the prior determined threshold of 75%. These observations suggest that the CAP does not require extensive training. However, the score results on the lingual and distal surfaces showed that the plasma plume could not circumvent anatomical constraints or overextended crowns that prevent proper access with the device. Nevertheless, the coronal and middle surface of the implant was sufficiently functionalised. Although the working area of the cold plasma plume is not limited to the visible part of the plume, which extends up to 8 mm length, we have to acknowledge, that effectivity of CAP is limited by the geometry of the defect and problematic accessibility.

Many studies have shown improved outcomes after training sessions with dental and medical students [6, 7] whereas the time series of this study showed no improvement in performance for treatment with the plasma device and only a moderate improvement with the waterjet. A possible explanation for this could be that the trainees were already experts, having at least 7 years of experience in periodontal or oral surgery. Therefore, they did not need intensive training, because these new devices did not require much different handling than traditional devices used in dentistry, such as laser or air polishing. In a dummy head trial, dental students were trained with two different systems to debride phantom teeth and it took the students 10 training sessions to reach the target [6]. Thus, non-experts may profit from systematic training with these instruments at the beginning of their dental career.

In this experiment, only the simple visual feedback was used as a learning method and repeated technical exercises were carried out to promote technical skills. This learning method proved to be sufficient to learn the handling with our devices, no advanced methods were needed [3]. As we conducted the training in the trainee`s hospital and had to travel across Germany with our equipment, simple, robust and quick to perform outcome measures were necessary. For the water jet, we felt confident that the visual control by the trainee was sufficient. For feedback with the plasma device, we chose the spreading of a water drop as a simple qualitative check for hydrophilicity.

This study also serves as a reminder that it is beneficial for practitioners to train on a dummy or extracted teeth when they buy a new device, even if it is very similar to devices used before.

A limitation of the present study is the artificial biofilm. A commercially available stain was used, that has similar limitations to the dyes used in the named air polishing studies. All stains represent a synthetic model of natural plaque and their adhesion properties differ from natural plaque. Some studies use permanent marker as dental plaque substitute [20, 21]. In our preliminary investigations, permanent marker (edding® 3000) adhered too strongly to rough titanium and showed little contrast to the dark grey implant surface. The artificial plaque used showed similar adhesion properties as microbial biofilms and showed a very high contrast to the implant surface, especially under UV-A light. This means that no colour threshold is needed for the analysis and a high detection rate of stain residues can be achieved. Another limitation is, that the camera was aligned perpendicular to the implant. Therefore, the angulations of 60° (upper aspect of the thread) and 120° (lower aspect of the thread) were not analysed separately. However, the deeper areas of the threads were generally more difficult to clean than the top thread surface areas. The set threshold of 70% stain removal was based on what experienced operators already familiar with the waterjet could achieve, rather than a real-world value. At the heart of all training courses is the question of whether the skills learned in a training can be transferred to real life. We hope to answer this question with our clinical trial. Only future clinical trials can tell us, what residual plaque means in terms of healing. As the focus of this study was on training, the possible influence of implant specific features such as thread depth, thread pitch, or thread design [22] was neglected.

## Conclusions

Training under simulated real-life conditions is important to become more familiar with the handling of the devices. The learning effect was very rapid from the first to the second treated implant, as the trainee received direct feedback on the “treatment success” immediately after the treatment. The efficiency of the water jet treatment varies depending on the accessibility to the implant. Three trainees had to repeat the training. Good treatment results with the cold plasma was constant from the beginning to the end. This training will help to ensure a greater consistency in the treatment of peri-implantitis in patients during our planned clinical trial.

## Data Availability

The datasets used and/or analysed during the current study are available from the corresponding author on reasonable request. The datasets generated and/or analysed for regulatory approval, which were not explicitly discussed in this study, are partially not publicly available due to international regulations.

## Appendix

Table

**Table 3:** Study characteristics. *N* = 172

Variable	Categories	*N* (%) or mean ± SD
Centre	Bonn	48 (27.9%)
	Frankfurt am Main	48 (27.9%)
	Greifswald	28 (16.3%)
	Kiel	48 (27.9%)
Light exposure	Low exposure	16 (9.3%)
	Normal exposure	52 (30.2%)
	Over-exposure	104 (60.5%)
Stain thickness	Nearly complete	120 (69.8%)
	Possibly insufficient	52 (30.2%)
Implant number	1	44 (25.6%)
	2	40 (23.2%)
	3	44 (25.6%)
	4	44 (25.6%)
Surface	Buccal	43 (25.0%)
	Mesial	43 (25.0%)
	Distal	43 (25.0%)
	Lingual	43 (25.0%)
Wettability score*		2.78 ± 0.43
Mean grey value of the image before treatment		107.8 ± 26.6
Mean grey value of the image after treatment		30.5 ± 23.4
Change in mean grey value of the image		77.3 ± 30.4
Mesial surfaces		
Mean grey value of the image before treatment		108.2 ± 27.1
Mean grey value of the image after treatment		20.2 ± 12.8
Change in mean grey value of the image		88.0 ± 26.9
Buccal surfaces		
Mean grey value of the image before treatment		109.3 ± 26.7
Mean grey value of the image after treatment		15.1 ± 10.8
Change in mean grey value of the image		94.3 ± 26.5
Distal surfaces		
Mean grey value of the image before treatment		106.1 ± 26.7
Mean grey value of the image after treatment		47.5 ± 27.7
Change in mean grey value of the image		58.5 ± 28.0
Lingual surfaces		
Mean grey value of the image before treatment		107.7 ± 26.8
Mean grey value of the image after treatment		39.2 ± 21.3
Change in mean grey value of the image		68.4 ± 26.4

*N*, number; *SD*, standard deviation. * values not available for one examiner from Greifswald (*N* = 160)

3

Table

**Table 4:** Distribution of the score measured for water drops on implants after cold plasma treatment discriminated between wettability score 0 to 3 (from coronal to apical implant area)

Drop position	Drop number	Score		Reached this score	
Coronal	1	0.0	0.6%		
		0.5	0.0%		
		1.0	1.1%	> 0.5	99.4%
Middle	2	1.5	3.4%	> 1.0	98.3%
		2.0	6.3%	> 1.5	94.9%
Apical	3	2.5	15.9%	> 2.0	88.6%
		3.0	72.7%	> 2.5	72.7%

4
